# Skeletal muscle microvascular hemodynamic responses during hyperinsulinemic-euglycemic clamp in a Zucker Diabetic Sprague Dawley rat model of type 2 diabetes

**DOI:** 10.3389/fphys.2025.1568145

**Published:** 2025-04-23

**Authors:** Gaylene M. Russell McEvoy, Brenda N. Wells, Meghan E. Kiley, Hamza Shogan, Graham M. Fraser

**Affiliations:** Division of BioMedical Sciences, Memorial University, St. John’s, NL, Canada

**Keywords:** oxygen mediated blood flow regulation, type 2 diabetes, zucker diabetic sprague dawley rat, capillary hemodynamics, microvascular blood flow

## Abstract

**Objective:**

We sought to measure skeletal muscle microvascular hemodynamic responses in Sprague Dawley (SD) and Zucker Diabetic Sprague Dawley (ZDSD) rat model of type 2 diabetes (T2D) at rest and during a hyperinsulinemic-euglycemic clamp under resting conditions and during acute changes in local tissue oxygen concentration [(O_2_)].

**Methods:**

Male SD and ZDSD rats were fed a high-fat diet, transitioned to a high-fat high-sugar diet from 16–19 weeks old to induce T2D in the ZDSD strain, then returned to the high-fat diet until intravital video microscopy (IVVM). At 27 weeks of age animals were fasted overnight, and on the morning of the IVVM experiment animals were anaesthetized, instrumented, and mechanically ventilated. The extensor digitorum longus muscle was blunt dissected, isolated, and reflected over a glass coverslip or a gas exchange chamber (GEC) fitted in the stage of an inverted microscope. Microvascular hemodynamic responses were recorded during baseline and hyperinsulinemic-euglycemic clamp without perturbation (Protocol 1) and during sequential changes in GEC [O_2_] (7%-12%-2%-7%) (Protocol 2).

**Results:**

In protocol 1, SD rats had a significant increase in red blood cell (RBC) velocity, RBC supply rate (SR), and RBC oxygen saturation (SO_2_) between baseline and euglycemia. However, ZDSD animals had no significant difference in hemodynamic responses and RBC SO_2_ between baseline and during hyperinsulinemic-euglycemic clamp. RBC SO_2_ was significantly higher in ZDSD than SD rats at baseline. In protocol 2, ZDSD rats had significantly higher RBC SO_2_ than their SD counterparts at 7% and 2% [O_2_]. RBC velocity, SR and capillary hematocrit showed no change from 7% in response to increased or decreased [O_2_] in either animal group. ZDSD rats had a significant increase between baseline and clamp in RBC SR at 12% as well as at 2% GEC [O_2_].

**Conclusion:**

SD rats had a robust increase in capillary hemodynamics during hyperinsulinemic-euglycemic clamp whereas the capillary hemodynamics in ZDSD rats did not significantly change. Additionally, SD and ZDSD rats lacked expected hemodynamic responses in response to local [O_2_] changes during baseline and hyperinsulinemic-euglycemic clamp. This finding suggests that hyperglycemia in T2D and high-fat feeding alter microvascular hemodynamic responses to acute changes in muscle [O_2_].

## 1 Introduction

Prevalence of type 2 diabetes (T2D) continues to mount worldwide, with an estimated 46% of Americans living with diabetes or pre-diabetes, 90%–95% of those diagnosed with diabetes have T2D. Diabetes represents a considerable economic burden amounting to approximately $412 billion in 2022 alone (Dall et al., 2019; Parker et al., 2024). Clinical diagnostic criteria for T2D includes insulin resistance, non-autoimmune decreased insulin production from β-cells of the pancreas, and hyperglycemia (ElSayed et al., 2023). Risk factors for T2D include obesity, inactivity, unhealthy diet, and increased age. T2D is characterized by insulin resistance and hyperglycemia (ElSayed et al., 2023). If left untreated, T2D has been shown to progress to nephropathy, neuropathy, retinopathy, and lead to an increased risk of cardiovascular disease (Casanova et al., 2017; Tien et al., 2017; Tuttle et al., 2005; Veves et al., 1998). Insulin binds to receptors on the endothelial cells of blood vessels, which activates endothelial nitric oxide synthase and produces nitric oxide (NO) causing vasodilation and increasing blood flow (Clerk et al., 2004; Kanai et al., 1993; King et al., 1983; Richey, 2013; Steinberg et al., 1994; Williams et al., 2018). Conversely, insulin binding to its receptor also results in the production of endothelin-1 (ET-1) leading to vasoconstriction. The balance between vasodilation and vasoconstriction is tightly controlled under healthy states; however, the balance is shifted towards vasoconstriction in human T2D (Fu et al., 2021; McVeigh et al., 1992; Motawi et al., 2013; Ren et al., 2017).

Obesity and a high-fat diet both contribute to the development of T2D in humans (Ruze et al., 2023). Human studies have identified impairment in O_2_ utilization during exercise, a reduced exercise capacity, slower V̇O_2_ kinetics, and lower V̇O_2_ in individuals with elevated BMI and T2D (Bauer et al., 2007; Green et al., 2007; Regensteiner et al., 2015). Directly measuring microvascular blood flow in human T2D patients remains out of reach based on current methodologies. Researchers have employed the use of several T2D rodent models to better understand the differences in blood flow in both healthy and diseased conditions. Vascular reactivity has been studied *in vivo* using the cremaster muscle and *ex vivo* techniques with cerebral arteries, and aortic rings in Zucker obese rats, Zucker Diabetic Fatty (ZDF), and Goto-Kakizaki (GK) T2D model rats (Brooks et al., 2015; Frisbee et al., 2016; Frisbee et al., 2019; Frisbee et al., 2011; Halvorson et al., 2019; Oltman et al., 2006; Phillips et al., 2005). Collectively, these works have determined that there are impairments in the vascular responses to stimuli in models of metabolic syndrome and T2D. Previous studies on the GK rat model of T2D have shown that the baseline capillary red blood cell (RBC) velocity and flux are lower than the Wistar controls indicating a decreased capacity for O_2_ delivery and transport (Padilla et al., 2006; 2007). GK rats exhibit a decrease in vascular density, impaired endothelial function, and impaired vascular reactivity (Frisbee et al., 2019; Sandu et al., 2000; Witte et al., 2003). Pre-diabetic ZDF rats also displayed reduced capillary RBC velocity, supply rate, and oxygen saturation (SO_2_) compared to controls (Ellis et al., 2010). Further, *in silico* models have described heterogeneous blood flow in striated muscle tissue to be a component in muscle oxygenation and endothelial function of metabolic syndrome and T2D (Frisbee et al., 2011; McClatchey et al., 2017; Sove et al., 2017). Studying vascular reactivity to local perturbations of skeletal muscle in high-fat diet fed healthy and T2D rodent models remains poorly understood.

Recently, the Zucker Diabetic Sprague Dawley (ZDSD) rat model of T2D has been developed as a model of human T2D (Peterson et al., 2015). ZDSD animals display a pre-diabetic phenotype from 15 to 22 weeks of age, the diabetic phenotype from 23 to 31 weeks of age with complications associated with advanced disease beyond 31 weeks of age (Han et al., 2020). The coordinated progression of T2D in the ZDSD strain is dependent on the dietary change to a high-sugar, high-fat diet from 16 to 19 weeks of age. The ZDSD model is advantageous for this study as the model mimics human disease progression with an intact leptin receptor and slower T2D disease progression. Additionally, the SD rats as a control strain, which facilitates comparing the control groups with previous studies that follow the same dietary regimen. Previous works have employed the use of hyperinsulinemic-euglycemic clamp to evaluate the level of insulin resistance in other T2D models while the effects of this procedure on skeletal muscle microvascular hemodynamics in the ZDSD rat model have yet to be evaluated.

Capillaries provide the major site of exchange of oxygen and nutrients, including glucose, to the tissue while simultaneously removing waste products and CO_2_. Regulation of blood flow to capillary beds is regulated by vasoconstriction and vasodilation of arterioles upstream that rely on signalling from downstream vessels in conjunction with local sensing and signalling (Segal, 2005). In healthy states, blood flow distribution to capillary beds is tightly controlled to ensure adequate supply of blood flow to downstream tissues thus matching the local metabolic demand (Murrant and Fletcher, 2022). It has previously been shown that there is a significant increase in capillary RBC velocity (Akerstrom et al., 2020) and supply rate in response to hyperinsulinemic clamp in healthy animals (Wells et al., 2023); however, this has yet to be measured in T2D models. Skeletal muscle capillary blood flow responses to altered [O_2_] via a gas based microfluidic device have been quantified previously; however, this work has focused on responses in young healthy rats (Ghonaim et al., 2013; Ghonaim et al., 2011; Russell McEvoy et al., 2022; Sove et al., 2021; Wells et al., 2023). Previous work with GK rat model of T2D has shown there is lower driving pressure to move O_2_ to the muscle and there is impaired O_2_ extraction in response to electrical muscle stimulation (Frisbee et al., 2019; Padilla et al., 2007). Development of rodent models for T2D and obesity have enabled the evaluation of microvascular blood flow and vascular reactivity during stages of disease progression. Coupling changes in local [O_2_] with hyperinsulinemic-euglycemic clamp in young healthy animals has demonstrating that fixing local muscle PO_2_ eliminates the insulin mediated hyperemia, providing evidence of an oxygen dependent component of the response (Wells et al., 2023); however, the impacts of coupling hyperinsulinemic-euglycemic clamp and O_2_ has yet to be evaluated in a T2D rodent model.

The purpose of our study was to quantify the skeletal muscle microvascular hemodynamics in the ZDSD rat model of T2D at rest and during a hyperinsulinemic-euglycemic clamp. Our first aim we sought to collect biometric data to obtain information about the time course progression of hyperglycemia and blood pressure in the ZDSD strain. We hypothesize that chronic hyperglycemia in the ZDSD rat model of T2D causes blood flow dysfunction at baseline and a blunted capillary hemodynamic response to hyperinsulinemic-euglycemic clamp compared to the age and diet matched control Sprague Dawley (SD) rats (aim 1). Our second aim was to quantify the capillary hemodynamic responses to dynamic changes in [O_2_] in high-fat, high-sugar fed 27-week-old SD and ZDSD rats during hyperinsulinemic-euglycemic clamp. We hypothesize that ZDSD rats experiencing hyperglycemia will have a blunted capillary hemodynamic response to altered [O_2_] during baseline and hyperinsulinemic-euglycemic clamp compared to their diet and age matched SD control group.

## 2 Materials and methods

### 2.1 Animal housing, feeding, and conscious sampling

All animal protocols were approved by the Memorial University Animal Care and Use Committee. Male Sprague Dawley (n = 20) and Zucker Diabetic Sprague Dawley (n = 28) rats were used in this study and were received from Charles River Laboratories. Animals were housed in pairs or groups of 3 in the Health Science Centre Animal Care Facilities throughout the duration of the protocol. All animals were fed Purina 5008 (16.7% kcal fat, 13.68% kcal carbohydrates from glucose, sucrose, fructose, and lactose) (LabDiet, Richmond, IN, United States) upon arrival to 16 weeks of age, followed by ResearchDiets D12468 rodent chow (47.7% kcal fat, 20.7% kcal carbohydrates from sucrose) (Research Diets, Inc., New Brunswick, NJ, United States) until 19 weeks of age, and then transitioned back to Purina 5008 for the remainder of the study. Animals were provided with chow and water *ad libitum*.

#### 2.1.1 Protocol 1 biweekly sampling

Following 2 weeks of acclimatization a subset of 12 SD rats (six 7-week-old and six 9-week-old) as well as 18 ZDSD rats (ten 7-week-old and eight 9-week-old) underwent biweekly fasted blood glucose sampling and conscious blood pressure measurements. For blood pressure measurement, animals were placed into rodent restraining tubes (IITC Life Science Inc., Woodland Hills, CA, United States) and allowed to acclimate in a warming box maintained at 33°C. Once acclimated, a plethysmography tail cuff was placed near the base of the rats’ tail and recordings of systolic blood pressure were made over a period of 7 min as per the manufacturer instructions (Model BP60-38″, IITC Life Science Inc., Woodland Hills, CA). A minimum of 3 samples within 10 mmHg were recorded to ensure a consistent measurement. Biweekly blood glucose measurements were collected from the saphenous vein using a handheld Contour Next One blood glucometer test strip (Ascensia Diabetes Care, Mississauga, ON, Canada).

#### 2.1.2 Protocol 2 biweekly measures

Fifteen-week-old SD (n = 8) and ZDSD (n = 10) rats were received from Charles River Laboratories and were fed Purina 5008 and D12468 diets as previously described until the intravital video microscopy (IVVM) experiment. Fasting blood glucose levels of ZDSD animals were measured between 16 and 19 weeks of age, a blood glucose greater than 15 mM was considered to be hyperglycemic. Animals were resampled biweekly up to 3 times, if blood glucose readings were not greater than 15 mM then they were excluded from the hyperglycemic cohort.

### 2.2 Intravital video microscopy preparation

**FIGURE 1:**
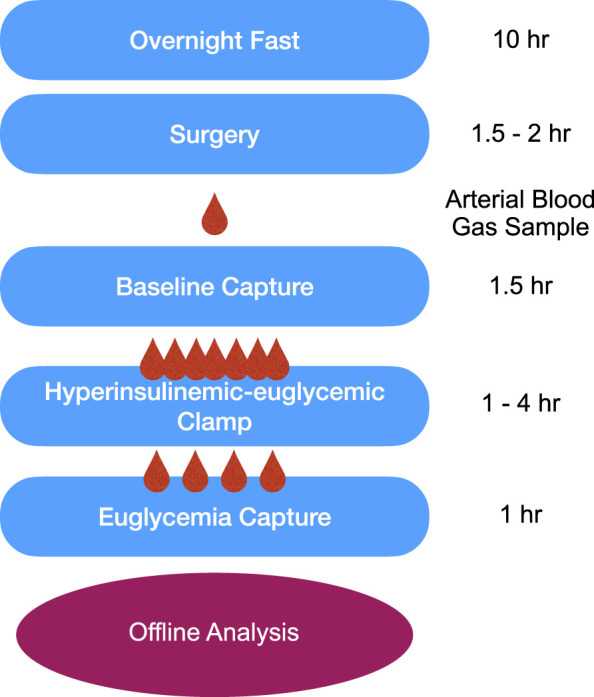
Timeline of the intravital video microscopy experimental protocol. Following an overnight fast, surgical preparation, and reflection of the extensor digitorum longus muscle on the microscope stage, animals were allowed to equilibrate for 30-min prior to data collection. Baseline 10X intravital videos were recorded followed by the hyperinsulinemic-euglycemic clamp. HumulinR was continuously infused at 2 U/mL/kg with simultaneous 50% glucose infusion at variable rates to achieve a stable blood glucose measurement between 5 and 7 mmol/L, as measured from a mixed tail clip blood sample. After reaching euglycemia, EDL intravital videos were recorded to allow comparison between the two conditions. All data was analyzed offline using custom MATLAB software.

Animals were fasted 10 h prior to IVVM surgical preparation. Animals were anesthetized with an intraperitoneal injection of sodium pentobarbital (65 mg/kg) and with additional amounts administered in larger animals (up to 100 mg/kg) as needed to effect (Euthanyl, Bimeda-MTC, Cambridge, Ontario, Canada). A timeline of intravital experiments is depicted in Figure 1. Upon establishing a surgical plane of anaesthesia as verified by the absence of toe pinch withdrawal and palpebral reflexes, a rectal temperature probe was inserted, and a heating pad and lamp were used to maintain the animals between 36°C and 37°C. Several 0.01 mL subcutaneous injections of lidocaine (20 mg/mL, Teligent Inc., Mississauga, ON, Canada) were introduced along the midline of the neck between the jaw and the sternum to ensure adequate local analgesia prior to vascular access.

The left common carotid artery was cannulated with polyethylene 50 tubing (BD Intramedic, Sparks, MD, United States; inner diameter 0.58 mm, outer diameter 0.965 mm) for continuous monitoring and recording of the animals’ systemic cardiovascular status. The right jugular vein was subsequently isolated from surrounding tissue and cannulated with a silastic tipped double cannula (Dow Corning, Midland, MI, United States; 0.635 mm outer diameter) to provide fluid resuscitation with 1 U/mL heparinized saline (2 mL/kg/hr), as well as to administer insulin and glucose during hyperinsulinemic-euglycemic clamp. Maintenance doses of pentobarbital (22 mg/kg) were administered intravenously at ∼30 min intervals, when the animal’s MAP exceeded 110 mmHg, or if the animal exhibited palpebral or corneal reflex. Animals were tracheotomized and mechanically ventilated with a mixture of ∼30% O_2_ and 70% N_2_. Ventilation rates and volumes were calculated based on the animal’s weight according to the ventilator manufacturer’s instructions (Inspira ASV, Harvard Apparatus, Holliston, MA, United States). Following completion of the tracheotomy, the neck incision was sutured closed with a continuous lock stitch.

The right extensor digitorum longus (EDL) muscle of the lower hind limb, was blunt dissected and isolated as previously described (Fraser et al., 2012; Tyml and Budreau, 1991). The distal tendon was cut near the retinaculum before the muscle was lifted and cleared from the surrounding tissue without damaging the feed artery, vein, and associated nervous innervation. The animal was then transferred onto the stage of an Olympus IX73 inverted microscope (Olympus, Tokyo, Japan). During protocol 1 the EDL was reflected over top of a glass coverslip, tethered at a length and tension approximate to that observed *in situ*, covered with a piece of polyvinylidene chloride film (Saran, Dow Corning Canada, Toronto, ON, Canada), and bathed in sterile saline warmed to 37°C. For protocol 2, the EDL was reflected over a 3D printed gas exchange chamber as previously described (Sove et al., 2021). The EDL was interfaced with a 50 µm thick oxygen permeable polydimethylsiloxane (PDMS) membrane (Dow Corning, Midland, MI, United States) separating the muscle from the gas channel (Sove et al., 2021). The gas conditions within the chamber were precisely controlled by mass flow controllers (SmartTrak100, Sierra Instruments, Monterey, CA, United States) coupled with a custom MATLAB user interface. As above, the muscle was covered in a piece of impermeable film (Saran, Dow Chemical Canada Inc., Toronto, ON, Canada) to isolate the muscle from room air. The muscle was gently compressed with a standard 22 mm glass coverslip with beads of vacuum grease (Dow Corning, Midland, MI, United States) applied along two opposing edges. Following the stage setup, animals were allowed to equilibrate for 30 min or until the animal’s temperature was between 36°C and 37°C and MAP was above 80 mmHg. Post acclimation, a 0.5 mL arterial blood sample was collected and loaded into a CG4+ cartridge and analyzed using a handheld VetScan iSTAT (Abbott Point of Care Inc., Princeton, NJ, United States). The blood gas analyzer provided measurements for pH, partial pressure of carbon dioxide (PCO_2_), partial pressure of oxygen (PO_2_), base excess in the extracellular fluid compartment concentration (BEecf), bicarbonate (HCO_3_) concentration, total carbon dioxide (TCO_2_), oxygen saturation (SaO_2_), and lactate concentration.

#### 2.2.1 Protocol 1: microvascular hemodynamic recordings

The EDL was transilluminated using a 300 W xenon arc light source (Sutter Lambda LS-OF30, San Francisco, CA, United States), transmitted via beam splitter (Optosplit II Bypass, Cairn, Kent, UK) equipped with 420 nm and 438 nm wavelength bandpass filters to simultaneously capture parfocal oxygen-dependent (438 nm) and isosbestic (420 nm) images using a Hamamatsu OrcaFlash4.0 v3 camera (Hamamatsu, Japan). Recordings were made using HCImage Live software (Hamamatsu, Japan) on a desktop computer. Each recording was 60 s in length and was captured at 30 frames per second.

An extended depth of field (EDF) map of the muscle surface was recorded by slowly focusing from the surface of the muscle to a depth of 100 µm into the tissue and then back to the surface of the muscle. Following completion of the EDF map 1-min captures of multiple focal planes of ten times magnification (×10) microscopic fields were recorded to quantify the baseline capillary flow in the EDL muscle. Recordings were only completed when the animals’ MAP was between 90 and 110 mmHg, and temperature was between 36°C and 37°C. A hyperinsulinemic-euglycemic clamp was completed as previously described (Leonard et al., 2005; Wells et al., 2023). Once euglycemia was achieved, IVVM recordings were repeated at the same areas that were imaged during baseline conditions to quantify the changes in skeletal muscle hemodynamics before and during hyperinsulinemic-euglycemia.

#### 2.2.2 Protocol 2: oxygen oscillation microvascular hemodynamic recordings

Using the same imaging system as Protocol 1, videos of microvascular blood flow were recorded using HCImage software (Hamamatsu, Japan) at 30 frames per second for 1 or 4 min as detailed below. Areas of microvascular blood flow were selected based on the number of in-focus capillaries, topology of the networks, and the brightness of the muscle area. Prior to imposing gas oscillations, a 1-min EDF recording was completed by focusing on the surface of the muscle and continuously changing the focal plane to capture all flowing vessels within the first 100 µm of the surface. A single focal plane within this tissue volume, and <60 µm of the surface of the muscle, was selected for the 4-min gas oscillation. Gas conditions imposed on the surface of the EDL muscle were designed to challenge the EDL muscle’s ability to respond to high (12%) and low (2%) O_2_ conditions. O_2_ oscillations were 4 min in duration and consisted of 7% O_2_ for 1 minute, 1 minute of 12% O_2_, 1 minute at 2% O_2_ followed by a return to 7% O_2_ for the remaining 1 minute. The CO_2_ concentration was set at 5% throughout the duration of the 4-min recording while N_2_ made up the balance of the gas composition. EDF recordings and gas oscillations were repeated three to five times in different areas across the muscle to best represent the flow state of the tissue.

### 2.3 Hyperinsulinemic-euglycemic clamp

A hyperinsulinemic-euglycemic clamp was completed as previously described (Leonard et al., 2005; Wells et al., 2023). Baseline blood glucose measures were determined from 3 blood samples taken from a tail clip using the handheld glucometer (Contour Next One, Ascensia Diabetes Care, Mississauga, ON, Canada). The infusion of insulin and glucose were administered via the double cannula which was inserted into the jugular vein. The hyperinsulinemic-euglycemic clamp was accomplished by infusing HumulinR (2 U/kg/min) (Eli Lilly Canada Inc., Toronto, ON, Canada) and variable rate of infusion of a 50% glucose (MilliporeSigma Canada Ltd., Oakville, ON, Canada) solution to titrate blood glucose to euglycemia based on repeated tail blood samples. Blood samples were collected every 5–10 min following the start of infusion to monitor blood glucose levels. Euglycemia was achieved when there were 3 stable tail measures between 5–7 mM, or arterial blood sample was within range. Video of microvascular networks previously recorded at baseline were repeated during euglycemia to be compared against baseline hemodynamic responses.

### 2.4 IVVM analysis and statistics

IVVM recordings of capillary blood flow were analyzed offline using custom MATLAB software (Ellis et al., 1990; Ellis et al., 1992; Ellis et al., 2012; Fraser et al., 2012; Japee et al., 2004) to quantify capillary hemodynamics including RBC velocity, supply rate (SR), and hematocrit. Capillary RBC SO_2_ was quantified as previously described (Ellis et al., 1990). Animals exhibiting abnormal microvascular hemodynamics, such as profound hyperemia (mean capillary RBC velocity >500 μm/s), at baseline or during hyperinsulinemic-euglycemic clamp were excluded from the presented data sets. One focal plane from each field of view was used to quantify functional capillary density (Bateman et al., 2001; Ellis et al., 2010; Fraser et al., 2015). In focus capillaries crossing test lines were ranked as continuous, stopped, or intermittent flow states; intermittent flow was defined as flow with stopped or reverse flow for >5 s of a 30 s recording (Bateman et al., 2001; Ellis et al., 2010; Fraser et al., 2015). Functional capillary densities were determined for each video by two independent observers using identical ranking criteria. All quantitative figures were generated using GraphPad Prism (GraphPad, V10.1.1, Boston, MA, United States).

A two-way analysis of variance (ANOVA) and a Tukey’s multiple comparisons *post hoc* test was conducted to compare weekly weight, biweekly blood glucose measurements between animal groups and ages. A one-way ANOVA and Tukey’s multiple comparisons test was used to compare blood glucose measures during hyperinsulinemic-euglycemic clamp and GIR between animal groups.

In protocol 1, a paired t-test was completed to compare hemodynamic parameter and RBC SO_2_ between baseline and hyperinsulinemic-euglycemic clamp for each group. A one-way ANOVA and Kruskal–Wallis *post hoc* test was done to determine significant differences between animal groups for hemodynamics and RBC SO_2_ measurements. A one-way ANOVA and a Tukey’s *post hoc* test was used to compare the proportion of continuous, intermittent, and stopped flow capillaries between animal groups. In protocol 2, capillary hemodynamic and SO_2_ data was compared using two-way ANOVA and Tukey’s multiple comparison tests between baseline and clamp values of each O_2_ conditions within each strain. Kruskal–Wallis and Dunn’s multiple comparisons tests were used to compare hemodynamic parameters and O_2_ condition between strains.

## 3 Results

### 3.1 Protocol 1: hyperinsulinemic-euglycemic clamp

#### 3.1.1 Conscious repeated measurements

**FIGURE 2:**
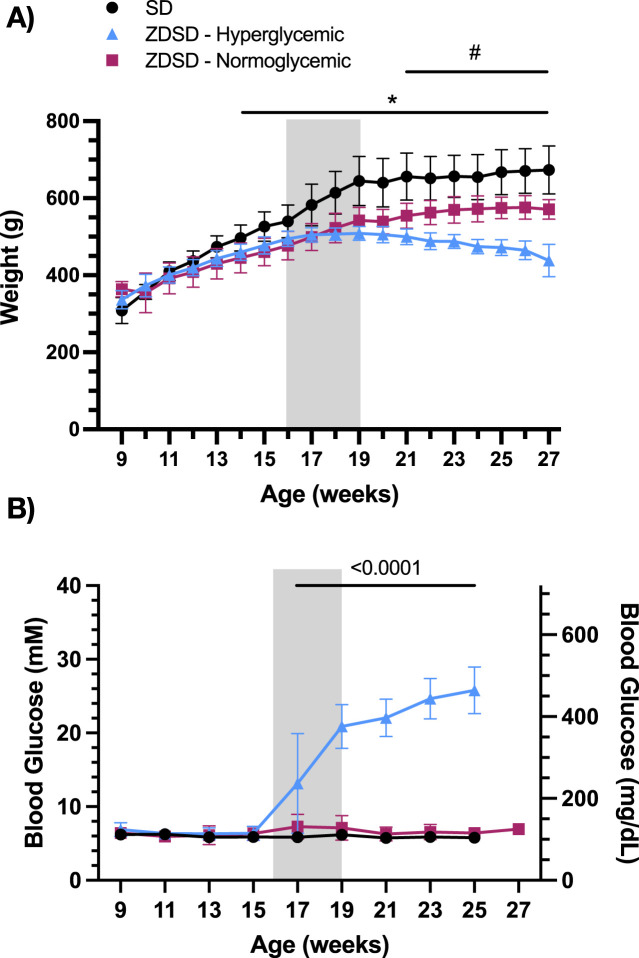
Sprague Dawley and Zucker Diabetic Sprague Dawley growth chart and blood glucose measurements from 9 to 27 weeks of age. Weekly morning body weight measures were recorded from 9 to 17 weeks of age **(A)**. Biweekly fasted saphenous blood glucose samples of Sprague Dawley (SD) and Zucker Diabetic Sprague Dawley (ZDSD) rats were collected biweekly from 9 to 27 weeks of age **(B)**. ZDSD animals were separated by blood glucose phenotype following completion of the intravital video microscopy experiment. Error bars represent standard deviation. Shaded region indicates period of high-fat high-sugar feeding. N = 10 SD, 5 normoglycemic ZDSD, 13 Hyperglycemic ZDSD rats. *p < 0.05 ZDSD compared to SD, #*p* < 0.05 hyperglycemic ZDSD compared to normoglycemic ZDSD.

Animal weights from 7 to 27 weeks of age were recorded weekly and are shown in Figure 2A. Hyperglycemic and normoglycemic ZDSD rats had significantly lower body weight compared to diet and age-matched SD rats from 14- to 27-weeks-old inclusive. Hyperglycemic ZDSD rats had significantly lower body weight compared to the normoglycemic ZDSD group from 21 to 27 weeks of age. Biweekly glucose samples from 9- to 25-weeks-old after a 6-h fast are shown in Figure 2B. ZDSD animals were stratified into normoglycemic, and hyperglycemic groups based on blood glucose measurements exceeding 15 mM at the time of the intravital video microscopy experiments. SD rats were normoglycemic (5–7 mM) throughout the duration of the study. The hyperglycemic ZDSD rats’ blood glucose was significantly elevated from 17- to 25-weeks-old compared to 9- to 15-week samples and are significantly elevated compared to normoglycemic and SD rats (Figure 2B).

Conscious biweekly systolic blood pressure measurements are reported in Table 1. Hyperglycemic ZDSD rats had significantly lower systolic pressures than their age matched SD controls at 9- and 13-weeks-old, the hyperglycemic group’s systolic pressures were significantly higher than the SD control group from 17- to 25-weeks-old. Hyperglycemic ZDSD rats also had significantly lower systolic blood pressure than the normoglycemic ZDSD group from 9 to 13 weeks of age. Additionally, at 15-, 17-, and 21- to 25-weeks-old ZDSD animals had significantly higher systolic blood pressure than the normoglycemic group. Normoglycemic ZDSD rats had significantly higher systolic blood pressure than the SD rats during all time points except at 15-weeks-old, at which time they were significantly lower.

**TABLE 1:** Conscious systolic blood pressure measurements from Sprague Dawley and Zucker Diabetic Sprague Dawley rats.

Age (weeks)	SD		ZDSD – Normoglycemic		ZDSD – Hyperglycemic	
	Mean ± StDev	N	Mean ± StDev	N	Mean ± StDev	N
9	145.3 ± 3.8	6	167.2 ± 3.9 *	3	140.5 ± 3.5 * #	7
11	161.3 ± 3.7	10	174.4 ± 2.6 *	5	162.8 ± 3.4 #	13
13	160.2 ± 3.5	10	169.3 ± 3.5 *	5	158.1 ± 2.6 * #	13
15	166.9 ± 2.6	10	164.5 ± 3.5 *	5	168.4 ± 2.9 #	13
17	166.8 ± 3.9	10	173.3 ± 2.1 *	5	179.9 ± 3.0 * #	13
19	170.8 ± 3.3	10	179.6 ± 2.5 *	5	178.8 ± 2.7 *	13
21	163.5 ± 3.2	10	168.0 ± 2.3 *	5	173.9 ± 3.2 * #	13
23	160.9 ± 2.9	10	177.0 ± 3.2 *	5	183.8 ± 3.5 * #	13
25	170.8 ± 2.9	10	171.0 ± 3.0	5	190.2 ± 2.2 * #	9

Note: SD, Sprague Dawley; ZDSD, Zucker Diabetic Sprague Dawley; StDev, Standard Deviation; N = number of animals. Variability in number of samples collected (N) is due to variable ages on arrival and no animals were sampled on the week of IVVM experiments (4 Hyperglycemic ZDSD). *p < 0.05 compared to SD, #*p* < 0.05 compared to normoglycemic ZDSD.

#### 3.1.2 Systemic measurements

Animal weight, age, and systemic animal monitoring data on day of IVVM are shown in Table 2. Animal weights were recorded prior to the anesthetic induction for IVVM experimental protocol. Mean, systolic, and diastolic blood pressures, and heart rate are based on the average of measures taken throughout the start of the baseline recording protocol and during the hyperinsulinemic-euglycemic clamp capturing protocol. Arterial blood gas measures from anaesthetized and ventilated rats are also recorded in Table 2. Systemic hematocrit values during the arterial sample and at the completion of the IVVM protocol are shown in Table 3. There are no significant differences in systemic hematocrit between the start and end of IVVM in the SD and normoglycemic ZDSD groups; however, the hyperglycemic ZDSD had significantly higher systemic hematocrit than SD rats at baseline (43.30% ± 2.49% vs. 46.25% ± 2.48%, *p* = 0.0178) and had significantly higher hematocrit during hyperinsulinemic-euglycemic clamp than during baseline (46.25% ± 2.48% vs. 49.20% ± 3.92%, *p* = 0.0384).

**TABLE 2:** Systemic animal data and arterial blood gas samples of anaesthetized male Sprague Dawley and Zucker Diabetic Sprague Dawley rats during protocol 1 intravital video microscopy.

	SD	ZDSD - normoglycemic	ZDSD - hyperglycemic
Age (weeks)	27.10 ± 0.32	27.8 ± 1.10	26.29 ± 0.95 ##
Weight (g)	672.90 ± 62.68	574.4 ± 23.89 ***	435.54 ± 18.42 **** ####
Mean Arterial Pressure (mmHg)	100.16 ± 2.74	99.30 ± 4.19	100.73 ± 3.30
Systolic Pressure (mmHg)	105.16 ± 2.79	107.59 ± 4.74	110.31 ± 5.19
Diastolic Pressure (mmHg)	92.55 ± 3.05	87.61 ± 3.37 *	89.21 ± 4.06
Heart Rate (beats/min)	303.36 ± 24.31	262.79 ± 6.80 **	265.69 ± 14.83 ***
pH	7.44 ± 0.05	7.38 ± 0.04 *	7.43 ± 0.03
PCO_2_ (mmHg)	39.62 ± 4.42	47.34 ± 6.06 **	43.98 ± 3.08
PO_2_ (mmHg)	110.1 ± 15.36	108.6 ± 6.02	110.3 ± 14.04
BEecf (mmol/L)	2.6 ± 2.27	2.4 ± 1.67	4.8 ± 2.23
HCO_3_ (mmol/L)	26.83 ± 1.84	27.64 ± 1,69	29.21 ± 1.92 *
TCO_2_ (mmol/L)	28.1 ± 1.85	29.2 ± 1.64	30.5 ± 1.97 *
SaO_2_ (%)	98.2 ± 0.92	97.8 ± 0.45	98.4 ± 0.50
Lac (mmol/L)	0.704 ± 0.60	2.294 ± 0.26 ****	0.776 ± 0.37 ####
N (animals)	10	5	13

Note: SD, Sprague Dawley; ZDSD, Zucker Diabetic Sprague Dawley; PCO_2_, partial pressure of carbon dioxide; PO_2_, partial pressure of oxygen; BEecf, base excess in the extracellular fluid compartment concentration; HCO_3_, bicarbonate concentration; TCO_2_, total carbon dioxide; SaO_2_, arterial oxygen saturation; Lac, lactate concentration. Values represent mean ± standard deviation. *p < 0.05, **p < 0.01, ***p < 0.001, ****p < 0.0001 compared to SD, ##*p* < 0.01, ####*p* < 0.0001 compared to normoglycemic ZDSD.

**TABLE 3:** Systemic hematocrit of Sprague Dawley and Zucker Diabetic Sprague Dawley rats.

	Start of IVVM	End of IVVM	N
SD	43.30 ± 2.49	43.56 ± 4.21	10
ZDSD - Normoglycemic	46.00 ± 1.22	45.63 ± 1.11	5
ZDSD - Hyperglycemic	46.25 ± 2.48 *	49.20 ± 3.92 #	12

Note: SD, Sprague Dawley; ZDSD, Zucker Diabetic Sprague Dawley; IVVM, Intravital Video Microscopy. Systemic hematocrit was not available for one hyperglycemic ZDSD, animal. Values represent mean ± standard deviation. *p = 0.0178 compared to SD, and #*p* = 0.0384 compared to start of IVVM.

#### 3.1.3 Blood glucose and glucose infusion rate

**FIGURE 3:**
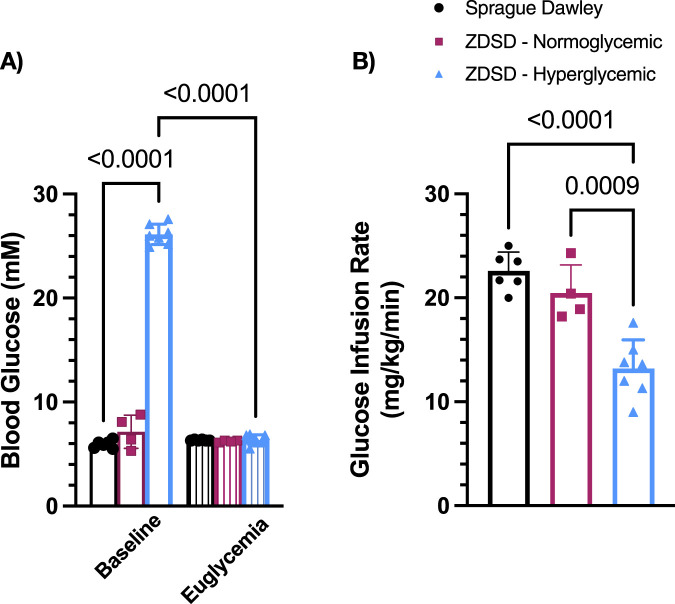
Blood glucose measurements and glucose infusion rates during hyperinsulinemic-euglycemic clamp in Sprague Dawley and Zucker Diabetic Sprague Dawley rats. Mean blood glucose **(A)** for each animal was determined using 3 sequential mixed blood samples from a tail clip. ZDSD animals were grouped by baseline blood glucose measurements during the intravital video microscopy experiment. Glucose infusion rates (GIR) were calculated based on mean infusion rate to achieve and maintain hyperinsulinemic-euglycemic clamp **(B)**. Bars represent mean ± standard deviation. N = 6 Sprague Dawley, 4 Normoglycemic Zucker Diabetic Sprague Dawley (ZDSD), and 7 Hyperglycemic ZDSD animals. P values are indicated in the figure, *p* < 0.05 were considered significant.

Blood glucose at baseline of hyperglycemic ZDSD were significantly higher than SD and normoglycemic ZDSD rats (26.14 ± 0.898 mM vs. 5.86 ± 0.416 mM, *p* = 0.0002, and 7.15 ± 1.59 mM, *p* < 0.0001, Figure 3A). Baseline hyperglycemic ZDSD blood glucose measurements were significantly different from paired measures during the hyperinsulinemic-euglycemic clamp (26.14 ± 0.90 mM vs. 6.24 ± 0.58 mM, *p* = 0.0134). There was no difference in blood glucose between baseline and at euglycemia in normoglycemic ZDSD rats (7.15 ± 1.59 mM and 6.23 ± 0.10 mM). Glucose infusion rates were significantly lower in hyperglycemic ZDSD than in SD rats (13.55 ± 2.76 mg/kg/min vs. 23.93 ± 3.92 mg/kg/min, *p* < 0.0001, Figure 3B) and significantly lower than the infusion rate required to achieve euglycemia for normoglycemic ZDSD (13.55 ± 2.76 mg/kg/min vs. 20.45 ± 2.72 mg/kg/min, *p* = 0.0009). Hyperglycemic ZDSD rats took significantly longer to reach hyperinsulinemic-euglycemic clamp than both SD and normoglycemic ZDSD (172.7 ± 33.71 min (110–202 min) vs. 72.85 ± 23.43 (46–142 min) and 83.50 ± 25.20 min (54–115 min), *p* < 0.0001).

#### 3.1.4 Capillary hemodynamic measurements

**FIGURE 4:**
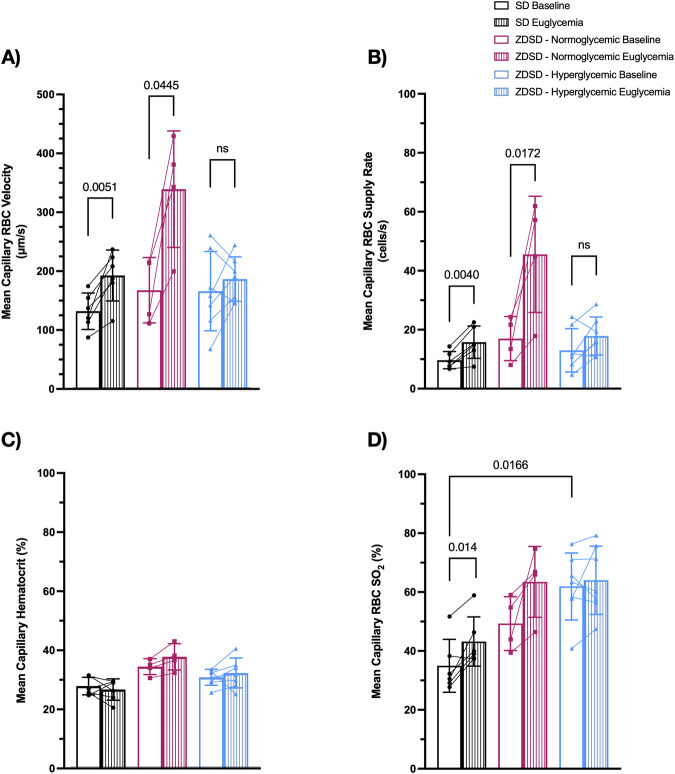
Capillary hemodynamics and red blood cell oxygen saturation of Sprague Dawley and Zucker Diabetic Sprague Dawley rats at baseline and during hyperinsulinemic-euglycemic clamp. Capillary RBC velocity **(A)**, RBC supply rate **(B)**, hematocrit **(C)**, and RBC SO_2_ **(D)**. Symbols correspond to per animal means. *p* < 0.05 were considered significant. N = 6 Sprague Dawley (SD), 4 Normoglycemic Zucker Diabetic Sprague Dawley (ZDSD), and 7 Hyperglycemic ZDSD animals.

Capillaries from 6 SD, 4 normoglycemic ZDSD, and 7 hyperglycemic ZDSD rats were selected for hemodynamic and RBC SO_2_ measurements. ZDSD animals were stratified into normoglycemic and hyperglycemic groups based on the measured blood glucose level at the beginning of hyperinsulinemic euglycemic clamp where hyperglycemia was determined to be >15 mM. There were significant increases in SD RBC velocity (131.3 ± 31.01 μm/s and 191.9 ± 43.17 μm/s, *p* = 0.0051, Figure 4A) and RBC SR (9.55 ± 2.93 cells/s and 15.68 ± 5.48 cells/s, *p* = 0.0040, Figure 4B) between baseline and euglycemic clamp. However, there is no significant difference in capillary hematocrit between baseline and euglycemia in SD rats (27.63% ± 2.97% and 26.46% ± 3.60%, Figure 4C). RBC SO_2_ significantly increased at clamp compared to baseline (34.93% ± 9.00% and 43.18% ± 8.36%, *p* = 0.0140, Figure 4D).

There was no significant difference in hyperglycemic ZDSD RBC velocity (175.6 ± 68.82 μm/s and 204.9 ± 64.51 μm/s, Figure 4A), RBC SR (12.90 ± 7.34 cells/s and 17.76 ± 6.45 cells/s, *p* = 0.0143, Figure 4B), hematocrit (30.59% ± 2.71% and 32.06% ± 5.06%, Figure 4C), and RBC SO_2_ (61.91% ± 11.40% and 64.00% ± 11.59%, Figure 4D). Normoglycemic ZDSD rats had significant increases in RBC velocity (166.8 ± 55.43 μm/s and 338.2 ± 98.87 μm/s, *p* = 0.0445, Figure 4A) and RBC SR (16.88 ± 7.49 cells/s and 45.48 ± 19.74 cells/s, *p* = 0.0172, Figure 4B). There were no significant differences in capillary hematocrit (34.20% ± 2.69% and 37.52% ± 4.43%, Figure 4C) and in RBC SO_2_ (49.28% ± 9.15% and 63.47% ± 12.02%, Figure 4D) between baseline and during clamp. RBC SO_2_ was significantly higher in the baseline hyperglycemic ZDSD group compared to the SD group (34.93% ± 9.00% and 61.91% ± 11.40%, *p* = 0.0166, Figure 4D). There were no significant differences in any other measures between the strains.

#### 3.1.5 Functional capillary density

**FIGURE 5:**
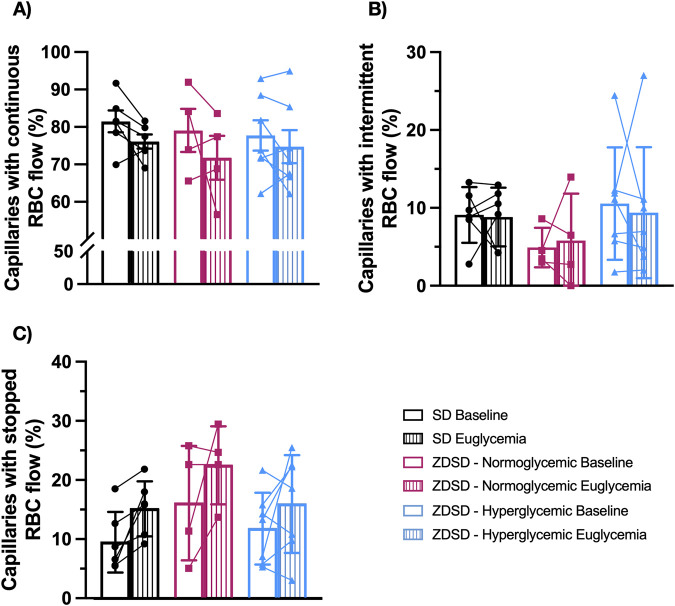
Functional capillary density measurements of Sprague Dawley and Zucker Diabetic Sprague Dawley rats. In focus capillaries with red blood cell (RBC) flow were determined to be continuous **(A)**, intermittent with reversed or stopped flow for >5 s **(B)** or stopped **(C)** flow at baseline and during hyperinsulinemic euglycemic clamp. Symbols correspond to per animal means before and during hyperinsulinemic-euglycemia. Bars represent mean. N = 6 Sprague Dawley (SD), 4 Normoglycemic Zucker Diabetic Sprague Dawley (ZDSD), and 7 Hyperglycemic ZDSD animals.

Functional capillary density measures of ZDSD and SD rats are shown in Figure 5. There were no significant differences in the proportion of continuously flowing, intermittent flowing, and stopped capillaries between SD and hyperglycemic ZDSD (continuous: 81.30% ± 7.18% vs. 77.55% ± 10.71%, intermittent: 9.11% ± 3.59% vs. 10.56% ± 7.23%, stopped: 9.59% ± 5.13% vs. 11.89% ± 6.09%). Similarly no differences were found between baseline and euglycemia for hyperglycemic ZDSD (continuous: 77.55% ± 10.71% vs. 74.55% ± 11.64%, intermittent: 10.56% ± 7.23% vs. 9.40% ± 8.43%, stopped: 11.89% ± 6.09% vs. 16.06% ± 8.28%), normoglycemic ZDSD group (continuous: 78.88% ± 11.52% vs. 71.59% ± 11.68%, intermittent: 4.91% ± 2.54% vs. 5.80% ± 6.05%, stopped: 16.21% ± 9.68% vs. 22.61% ± 6.60%) or SD (continuous: 81.30% ± 7.18% vs. 75.92% ± 4.59%, intermittent: 9.11% ± 3.59% vs. 8.83% ± 3.77%, stopped: 9.59% ± 5.13% vs. 15.25% ± 4.66%).

### 3.2 Protocol 2: hyperinsulinemic-euglycemic clamp with O_2_ oscillations

#### 3.2.1 Systemic animal data

Animal data including age, weight, and systemic data recorded during the IVVM experiment are shown in Table 4. Animal weights were collected prior to anaesthesia. Hyperglycemic ZDSD rats had significantly lower bodyweight than SD and normoglycemic ZDSD at the IVVM endpoint. Normoglycemic ZDSD were significantly lighter than SD controls at IVVM endpoint. Mean arterial pressure, systolic blood pressure, diastolic blood pressure, and heart rate are the averages from the duration of the IVVM recording period and include brief transient periods of lower blood pressure immediately following bolus anaesthetic administration. Arterial blood gas results for both SD and ZDSD rats are shown in Table 4.

**TABLE 4:** Systemic animal data and arterial blood gas samples of anaesthetized male Sprague Dawley and Zucker Diabetic Sprague Dawley rats during protocol 2.

	SD	ZDSD - normoglycemic	ZDSD - hyperglycemic
Age (weeks)	27.23 ± 0.51	27.21 ± 0.10	26.95 ± 0.43
Weight (g)	683.25 ± 52.01	597.50 ± 24.75 *	425.00 ± 14.36 **** ####
Mean Arterial Pressure (mmHg)	105.67 ± 4.70	102.20 ± 1.78	98.63 ± 5.21 *
Systolic Pressure (mmHg)	121.28 ± 5.94	121.74 ± 2.60	110.34 ± 5.56 **
Diastolic Pressure (mmHg)	90.17 ± 4.79	84.96 ± 1.16	85.82 ± 7.97
Heart Rate (beats/min)	331.95 ± 20.41	297.93 ± 9.91	250.79 ± 14.92 **** #
pH	7.45 ± 0.02	7.44 ± 0.01	7.39 ± 0.05 *
PCO_2_ (mmHg)	39.06 ± 1.12	41.60 ± 0.28	42.78 ± 3.25 *
PO_2_ (mmHg)	117.13 ± 15.38	101.00 ± 0.00	116.38 ± 10.45
BEecf (mmol/L)	3.25 ± 1.75	3.50 ± 0.71	1.38 ± 3.81
HCO_3_ (mmol/L)	27.23 ± 1.57	28.00 ± 0.71	26.24 ± 3.02
TCO_2_ (mmol/L)	28.63 ± 1.51	29.50 ± 0.71	27.38 ± 3.16
SaO_2_ (%)	98.63 ± 0.52	98.00 ± 0.00	98.43 ± 0.53
Lac (mmol/L)	0.52 ± 0.17	2.92 ± 0.27 ****	0.69 ± 0.61 ####
N	8	2	8

Note: SD, Sprague Dawley; ZDSD, Zucker Diabetic Sprague Dawley; PCO_2_, partial pressure of carbon dioxide; PO_2_, partial pressure of oxygen; BEecf, base excess in the extracellular fluid compartment concentration; HCO_3_, bicarbonate concentration; TCO_2_, total carbon dioxide; SaO_2_, arterial oxygen saturation; Lac, lactate concentration. Values represent mean ± standard deviation. *p < 0.05, **p < 0.01, ****p < 0.0001 compared to SD, #*p* < 0.05, ####*p* < 0.0001 compared to normoglycemic ZDSD.

#### 3.2.2 Hyperinsulinemic-euglycemic clamp

**FIGURE 6:**
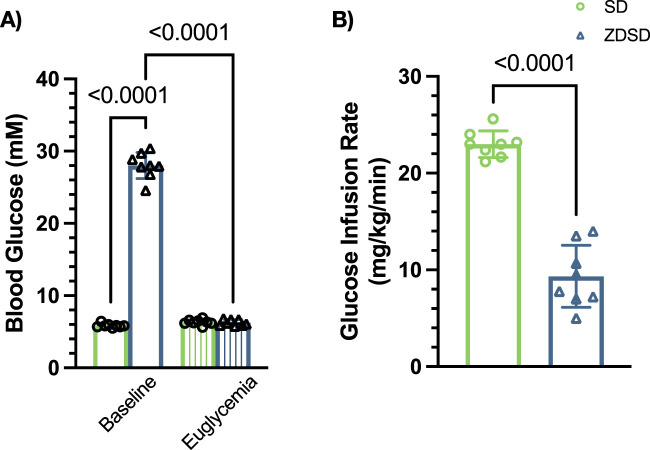
Blood glucose measure of Sprague Dawley and Zucker Diabetic Sprague Dawley rats before and during hyperinsulinemic-euglycemic clamp with oxygen perturbations. Each symbol represents average of 3 baseline measurements and 3 measures from each animal taken immediately preceding hyperinsulinemic-euglycemic clamp recording **(A)**. Glucose infusion rate (GIR), in mg/kg/min, was calculated beginning when euglycemia was achieved and throughout the duration of the hyperinsulinemic-euglycemic clamp recording period **(B)**. Bars represent mean ± standard deviation. N = 8 SD and 8 ZDSD animals. *p* values less than 0.05 were considered significant and are included in the figures.

The average of 3 baseline measures and 3 measures immediately preceding hyperinsulinemic-euglycemic clamp IVVM recordings are shown in Figure 6A. ZDSD rats had significantly higher blood glucose at baseline compared to SD control rats at baseline (28.01 ± 1.80 mM vs. 5.85 ± 0.272 mM, *p* < 0.0001). The blood glucose of ZDSD rats significantly decreased during hyperinsulinemic-euglycemic clamp (28.01 ± 1.80 mM vs 6.26 ± 0.424 mM, *p* < 0.0001). There were no differences between baseline and clamp values for SD rats (5.85 ± 0.272 mM vs 6.32 ± 0.380 mM, *p* = 0.7070). Sprague Dawley rats required a significantly higher glucose infusion rate, in mg/kg/min, to achieve euglycemia compared to hyperglycemic ZDSD rats (23.01 ± 1.38 vs 9.34 ± 3.21, *p* < 0.0001, Figure 6B). Due to hyperemic blood flow observed in one normoglycemic ZDSD, the IVVM data for the singular normoglycemic ZDSD rat was omitted from the presented data.

#### 3.2.3 Capillary hemodynamic responses to oxygen

**FIGURE 7:**
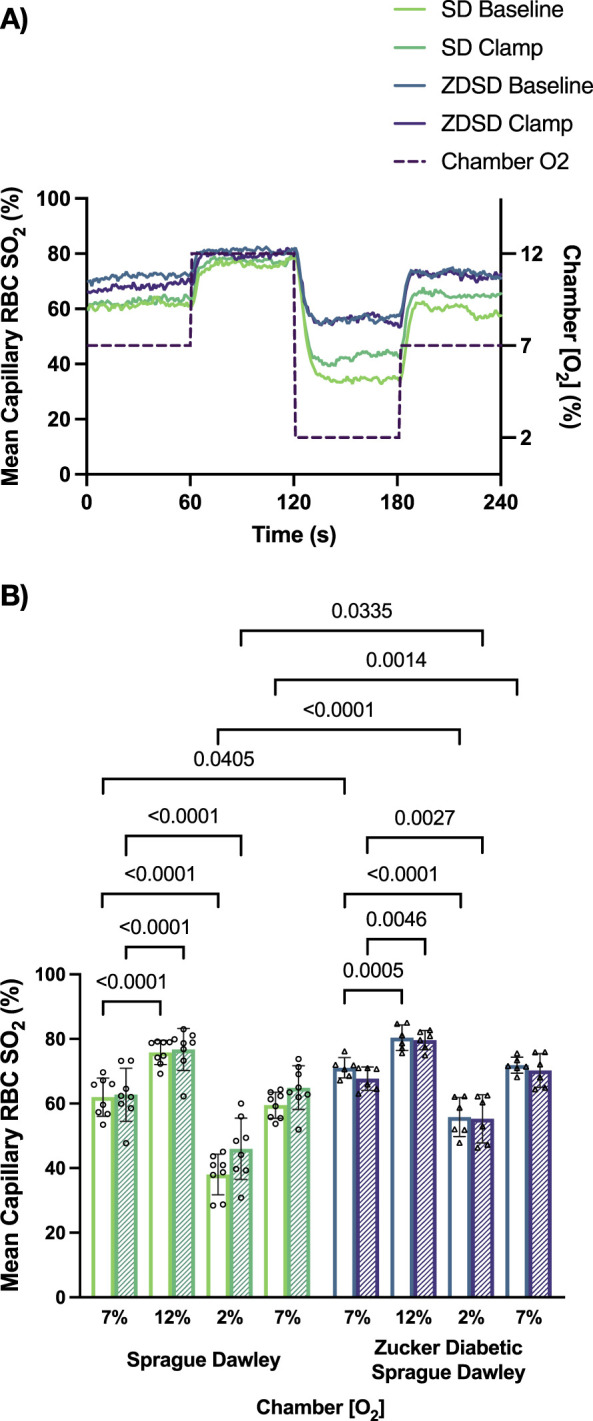
Mean capillary red blood cell oxygen saturation of Sprague Dawley and Zucker Diabetic Sprague Dawley rats in response to oxygen oscillations during baseline and hyperinsulinemic-euglycemic clamp. Time series of capillary red blood cell (RBC) oxygen saturation (SO_2_) (%) measurements from extensor digitorum longus muscle of Sprague Dawley (SD) and Zucker Diabetic Sprague Dawley (ZDSD) rats interfaced with a gas exchange chamber. Changes in oxygen concentration ([O_2_]) were oscillated from 7% to 12%–2% - 7% for 1 min each. Capillary data for each second from SD and ZDSD rats were calculated for 240s to create time transient plots **(A)**. **(B)** indicates the per animal mean values of the last 15 s of each gas condition at baseline (empty bars) and during hyperinsulinemic-euglycemic clamp (hatched bars). N = 8 SD animals, 132 capillaries at baseline, 100 capillaries during clamp. N = 6 ZDSD animals, 124 capillaries at baseline, and 59 capillaries during clamp. Bars represent mean ± standard deviation. *p < 0.05, **p < 0.01, ****p < 0.0001.

Capillary RBC SO_2_ measurements, in %, of SD and ZDSD rats in response to changes in [O_2_] and during baseline and hyperinsulinemic-euglycemic clamp are shown in Figure 7. SD rats had significantly higher RBC SO_2_ during 12% [O_2_] at baseline and clamp compared to the 7% [O_2_] condition (*p* < 0.0001, and *p =* 0.0001, respectively). RBC SO_2_ significantly decreased when the EDL was exposed to 2% [O_2_] compared to 7% at baseline and during hyperinsulinemic-euglycemia (*p* < 0.0001, and *p <* 0.0001, respectively). There was no significant difference in SD RBC SO_2_ between the first and second 7% [O_2_] at either time point (*p =* 0.9852 and *p* = 0.9971, respectively). No significant differences in RBC SO_2_ were observed between baseline and euglycemia during the first 7%, 12%, 2% and second 7% [O_2_] exposure (*p* > 0.9999, *p* > 0.9999, *p =* 0.1508, and *p* = 0.6348, respectively).

Similarly, ZDSD rats had significant increases in RBC SO_2_ at 12% [O_2_] compared to 7% at baseline and during hyperinsulinemic-euglycemic clamp (*p* = 0.0005 and *p* = 0.0046, respectively). Significant decreases in RBC SO_2_ were observed at 2% compared to 7% [O_2_] at baseline and euglycemia (*p* < 0.0001 and *p* = 0.0027). No differences were measured between the first and second 7% [O_2_] at either time point (*p* > 0.9999 and *p* = 0.9934). At each of the four [O_2_] concentrations there were no differences in ZDSD RBC SO_2_ between baseline and clamp with p-values ranging from 0.9183 to >0.9999. ZDSD rats had significantly higher RBC SO_2_ than SD rats during baseline at 7% (*p =* 0.0405), 2% (*p <* 0.0001), and the second 7% (*p =* 0.0014) as well as at clamp under 2% [O_2_] (*p =* 0.0335).

**FIGURE 8:**
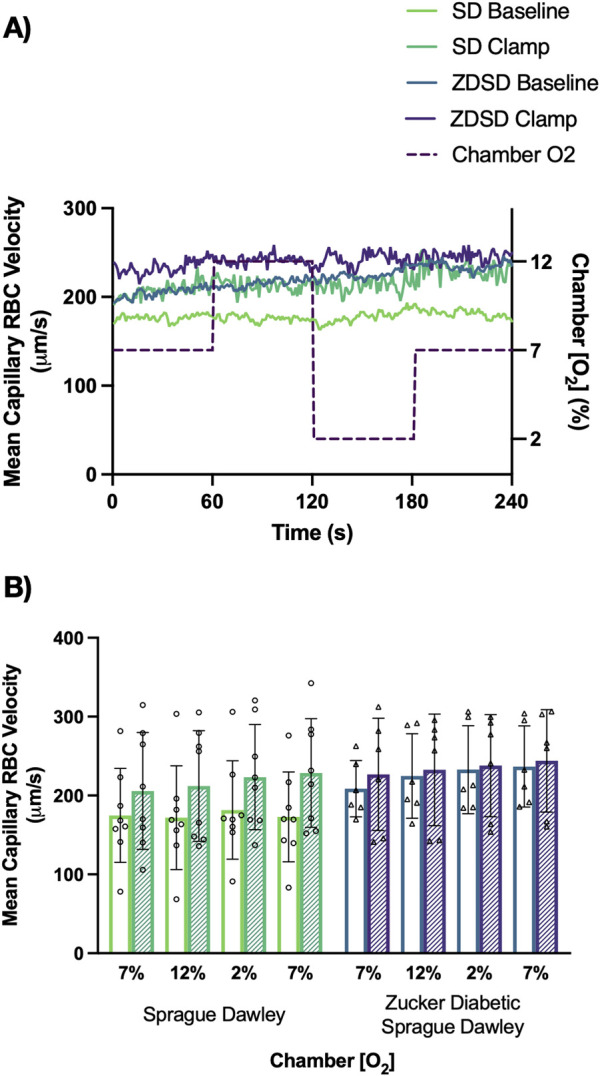
Mean capillary red blood cell velocity of Sprague Dawley and Zucker Diabetic Sprague Dawley rats in response to oxygen oscillations during baseline and hyperinsulinemic-euglycemic clamp. Time series of capillary red blood cell (RBC) velocity, in µm/s, measurements from extensor digitorum longus muscle of Sprague Dawley (SD) and Zucker Diabetic Sprague Dawley (ZDSD) rats interfaced with a gas exchange chamber. Changes in oxygen concentration [(O_2_)] were oscillated from 7% to 12%–2% - 7% for 1 min each. Capillary data for each second from SD and ZDSD rats were calculated for 240s to create time transient plots **(A)**. **(B)** indicates the per animal mean values of the last 15 s of each gas condition at baseline (empty bars) and during hyperinsulinemic-euglycemic clamp (hatched bars). N = 8 SD animals, 243 capillaries at baseline, 179 capillaries during clamp. N = 6 ZDSD animals, 225 capillaries at baseline, and 95 capillaries during clamp. Bars represent mean ± standard deviation.

Capillary RBC velocity responses to O_2_ oscillations during baseline and hyperinsulinemic-euglycemic clamp for SD and ZDSD rats are shown in Figure 8. SD rats had no significant changes in mean capillary RBC velocity between baseline and high [O_2_] or in response to low [O_2_]. Additionally, there was no change in RBC velocity between the first 7% and the second 7%. Similarly, during hyperinsulinemic-euglycemic clamp there were no significant differences in RBC velocity between 7%, 12%, and 2%. There was no significant difference in RBC velocity between baseline and during hyperinsulinemic-euglycemic clamp. ZDSD rats had no significant change in capillary RBC velocity response to changing [O_2_] from 7% to 12% and 2%. There was no difference between the first and second 7% [O_2_] conditions at baseline. During hyperinsulinemic-euglycemic clamp there were no significant differences between 7%, 12%, 2%, and the second 7% condition. When each [O_2_] was compared between baseline and hyperinsulinemic clamp there were no differences in RBC velocity for 7%, 12%, 2%, and the second 7%, *p-*values being equal to or greater than 0.09747. There were no significant differences between the RBC velocity measurements of SD and ZDSD animals when comparing their baseline [O_2_] conditions and their [O_2_] conditions during hyperinsulinemic-euglycemic clamp.

**FIGURE 9:**
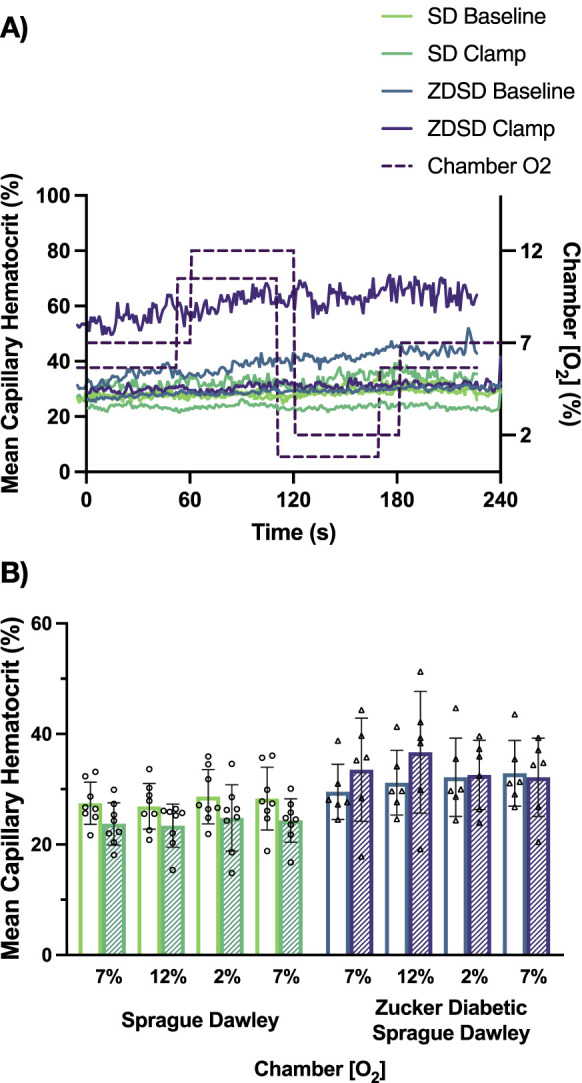
Mean capillary hematocrit of Sprague Dawley and Zucker Diabetic Sprague Dawley rats in response to oxygen oscillations during baseline and hyperinsulinemic-euglycemic clamp. Time series of capillary hematocrit (%) measurements from extensor digitorum longus muscle of Sprague Dawley (SD) and Zucker Diabetic Sprague Dawley (ZDSD) rats interfaced with a gas exchange chamber. Changes in oxygen concentration ([O_2_]) were oscillated from 7% - 12% - 2% - 7% for 1 min each. Capillary data for each second from SD and ZDSD rats were calculated for 240s to create time transient plots **(A)**. **(B)** indicates the per animal mean values of the last 15 s of each gas condition at baseline (empty bars) and during hyperinsulinemic-euglycemic clamp (hatched bars). N = 8 SD animals, 243 capillaries at baseline, 179 capillaries during clamp. N = 6 ZDSD animals, 225 capillaries at baseline, and 95 capillaries during clamp. Bars represent mean ± standard deviation.

Capillary hematocrit responses to hyperinsulinemia and [O_2_] changes are shown in Figure 9. SD rats had no significant differences between imposed gas chamber [O_2_] conditions at baseline or during hyperinsulinemic-euglycemic clamp with *p-*values being equal to or greater than 0.9742. When compared between baseline and hyperinsulinemic-euglycemic clamp, the first 7%, 12%, 2%, and the second 7% showed no significant difference between the 2 glycemic states. ZDSD rats had no significant responses to changes in [O_2_] at both baseline and during hyperinsulinemic-euglycemic clamp. Between baseline and clamp there were no significant differences at 7%, 12%, 2%, and the second 7% [O_2_]. SD and ZDSD rats had no significant differences in capillary hematocrit during baseline O_2_ oscillations.

**FIGURE 10:**
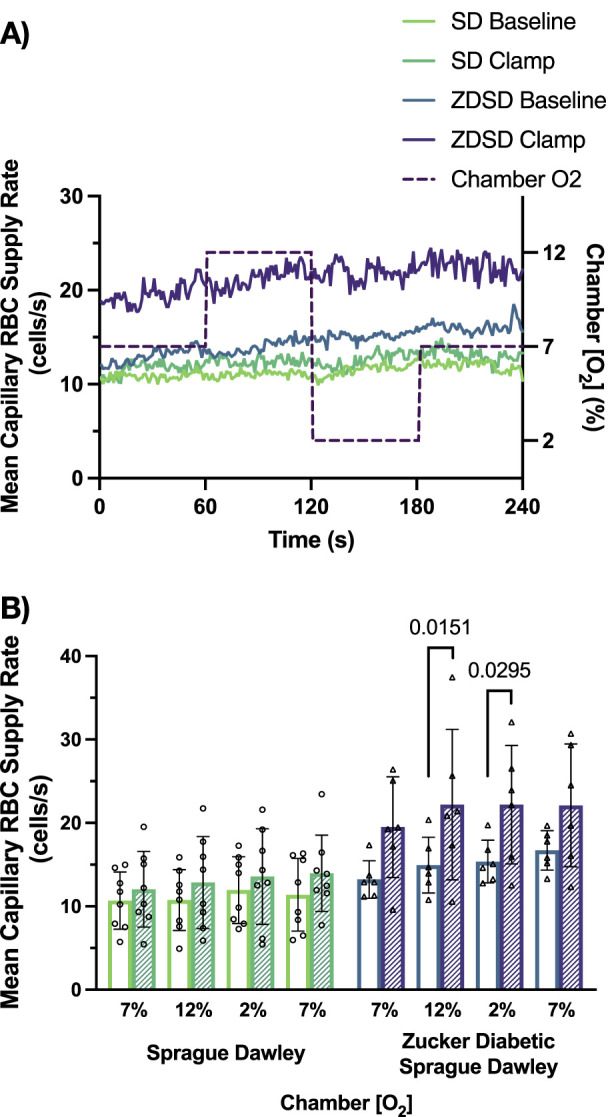
Mean capillary red blood cell supply rate of Sprague Dawley and Zucker Diabetic Sprague Dawley rats in response to oxygen oscillations during baseline and hyperinsulinemic-euglycemic clamp. Time series of capillary red blood cell (RBC) supply rate, in cells/s, measurements from extensor digitorum longus muscle of Sprague Dawley (SD) and Zucker Diabetic Sprague Dawley (ZDSD) rats interfaced with a gas exchange chamber. Changes in oxygen concentration [(O_2_)] were oscillated from 7% - 12% - 2% - 7% for 1 min each. Capillary data for each second from SD and ZDSD rats were calculated for 240 s to create time transient plots **(A)**. **(B)** indicates the per animal mean values of the last 15 s of each gas condition at baseline (empty bars) and during hyperinsulinemic-euglycemic clamp (hatched bars). N = 8 SD animals, 243 capillaries at baseline, 179 capillaries during clamp. N = 6 ZDSD animals, 225 capillaries at baseline, and 95 capillaries during clamp. Bars represent mean ± standard deviation. *p* values <0.05 were considered significant and are indicated in the figure.

Capillary RBC supply rate responses to hyperinsulinemic-euglycemic clamp and O_2_ perturbations are shown in Figure 10. SD rats had no significant changes in RBC SR between the 4 O_2_ conditions at baseline or during hyperinsulinemic-euglycemic clamp. There were no significant differences in RBC SR between baseline and hyperinsulinemic-euglycemic clamp at each gas condition. The capillary RBC SR at baseline in ZDSD rats was not significantly different from the 7% baseline condition at 12%, 2% or the second 7% [O_2_]. During hyperinsulinemic-euglycemic clamp a similar result was observed that there was no difference in SR in response to high or low [O_2_]. However, when baseline and hyperinsulinemic-euglycemic clamp SR were compared in ZDSD rats there was near significant increase in the first 7% [O_2_] condition (*p* = 0.0610) as well as significant increases during hyperinsulinemic-euglycemic in response to the 12% [O_2_] (*p* = 0.0151) and 2% [O_2_] (*p* = 0.0295). Similar to the first 7% [O_2_] the second 7% condition was elevated during clamp but not significantly (*p* = 0.1768). There were no significant differences between SD and ZDSD during any gas condition.

## 4 Discussion

The purpose of this study was to quantify the capillary hemodynamic response at baseline and during hyperinsulinemic-euglycemic clamp in the ZDSD rat model of T2D under stable conditions and during [O_2_] variations. Additionally, in this study we sought to collect biometric measures to quantify the development of chronic hyperglycemic in the ZDSD rat model. For the quantification of microvascular blood flow, intravital video microscopy was used to image and record capillary blood flow in skeletal muscle of male SD and ZDSD rats during baseline and a hyperinsulinemic-euglycemic clamp. To assess the responsiveness to altered [O_2_] in protocol 2, we employed a microfluidic gas exchange chamber that simultaneously allows for manipulation of oxygen concentration at the surface of the muscle and visualization of the microvascular blood flow. Functional capillary density was measured to compare relative proportional differences in capillaries with continuous, intermittent, and stopped red blood cell flow.

Biweekly fasted blood glucose measures verified that the development of chronic hyperglycemia in ZDSD animals was comparable with the phenotype of untreated human T2D. Interestingly, 5 and 2 ZDSD animals, from protocols 1 and 2 respectively, remained normoglycemic throughout the duration of the study demonstrating marked heterogeneity in the ZDSD hyperglycemic phenotype. Normoglycemic ZDSD animals were housed in identical conditions, provided the same access to the high-fat high-sugar diet, and were sampled at the same frequency as their SD control and ZDSD hyperglycemic counterparts. The variability in the hyperglycemic phenotype is not clearly documented in the literature as several studies report that 100% of animals develop hyperglycemia (Hammond et al., 2014; Peterson et al., 2015) whereas others also report that some subset of animals remain normoglycemic (Creecy et al., 2016; Davidson et al., 2014; Gonzalez et al., 2014; Han et al., 2020; Hill Gallant et al., 2014; Reinwald et al., 2009; Suckow et al., 2017). ZDSD rats are expected to develop hypertension at a later age due to the progression of T2D (Han et al., 2020; Jackson et al., 2015). The normoglycemic ZDSD group was not significantly different from the SD cohort at 25 weeks of age, which further suggests this group is a phenotypical intermediate between the normal control and the hyperglycemic groups. Hyperglycemic animals exhibited significant weight loss following the transition from the high-fat high-sugar diet to return to the high-fat Purina 5008. The decrease in body weight was also observed by Creecy et al. following the transition from the high-fat high-sugar diet back to Purina 5008 (Creecy et al., 2016). This decrease may be linked to the progression of T2D in hyperglycemic ZDSD animals as they begin to develop diabetic complications and potentially begin to lose of subcutaneous fat mass and experience muscle wasting.

Marked hyperglycemia in the ZDSD rats is consistent with T2D symptoms, which includes hyperinsulinemia prior to β-cell exhaustion. Although not measured in the current study, previous work has reported that ZDSD animals have elevated blood insulin by 13 weeks and exhibit substantial insulin resistance (Peterson et al., 2015). Ten to 11-week-old ZDF rats have shown impaired glucose tolerance in response to hyperinsulinemic-euglycemic clamp with glucose infusion rates of 1.8 and 3.3 mg/kg/min (Leonard et al., 2005; Shogan et al., 2019). However, our results show that hyperglycemic ZDSD rats uptake glucose at a higher rate in response to insulin than the GK and ZDF strains at earlier ages (Clerk et al., 2007; Elgebaly et al., 2008; Leonard et al., 2005; Shogan et al., 2019). The ZDSD strain has diminished insulin-mediated glucose uptake compared to their age matched SD group (Figures 3, 6) indicating that while the ZDSD rats experience hyperglycemia for several weeks and their insulin sensitivity has decreased, the downstream pathways to translocate GLUT4 within skeletal muscle remain at least partially functional. Diminished glucose uptake is not the sole cause of the hyperglycemic state observed in the ZDSD group. Impaired glucose uptake at the myocyte can be due to the delivery of glucose via the vasculature, glucose uptake into the myocyte, the intracellular metabolism/phosphorylation of glucose within the intracellular space, or a combination of these steps (Wasserman and Ayala, 2005). Future studies will have to consider the alteration in intracellular glucose handling as potential explanation for the hyperglycemic state in the ZDSD rat strain.

The rate of glucose uptake and the amount of blood delivered to the tissue determines the amount of glucose that skeletal muscle can take up. The mechanisms of insulin-mediated vasoreactivity are a key factor to consider when examining blood flow regulation in response to hyperinsulinemic-euglycemic clamp (Piatti et al., 1996; Steinberg et al., 1994). In healthy states, insulin leads to vasodilation via upregulation of NO production; however, in T2D insulin can cause vasoconstriction through the production of ET-1 (Haak et al., 1992; Olver et al., 2019; Takahashi et al., 1990). *Ex vivo* studies have shown prolonged insulin exposure leads to an increase in ET-1 signaling and decreased vasodilation. However, in this study there were no significant increases nor decreases in RBC velocity, RBC SR, nor capillary hematocrit between hyperglycemic ZDSD, normoglycemic ZDSD, or the SD control group during baseline (Figures 4A–C, respectively). This finding was not consistent with the literature findings using GK rats where baseline capillary blood flow was significantly lower than skeletal muscle blood flow of Wistar control rats (Padilla et al., 2006). Padilla and colleagues have suggested that the decrease in blood flow they observed may be due to glycosylation of RBC membranes, hyperglycemia altering the glycocalyx, or an increase in ET-1 production. Our findings suggest that the overall supply of red cells to tissues is not impaired; however, when considering RBC SO_2_ at the capillary level, baseline O_2_ saturation is significantly higher in the hyperglycemic group than in the SD animals indicating a mismatch in O_2_ supply and demand (Figure 4D). Our SD group also demonstrated lower RBC SO_2_ (34.9% ± 9.0% during baseline) than the RBC SO_2_ measured in young healthy SD rats (42.8% ± 4.6%) (Wells et al., 2023). Interestingly, the baseline RBC SO_2_ of normoglycemic ZDSD rats exhibit an intermediate condition between the other 2 groups possibly indicating an O_2_ supply-demand mismatch, as indicated by RBC SO_2_, in the hyperglycemic group may be due to strain but further enhanced by hyperglycemia.

ZDSD rats had impaired capillary hemodynamic response to hyperinsulinemic-euglycemic clamp compared to the diet and age matched SD group. We hypothesized there would be a significant increase in RBC velocity and RBC SR in the SD cohort while we expected a decrease in RBC SR and velocity in the hyperglycemic ZDSD group. These hypotheses were based on the previously reported effects of insulin on vasodilation and vasoconstriction (Baron et al., 1995; Cleland et al., 1999; Miller et al., 2002) and works that identified impaired vasodilation of arterioles in T2D (Frisbee et al., 2019; Haak et al., 1992; Miller et al., 2002; Takahashi et al., 1990). Our findings demonstrate that although the RBC supply and velocity did increase during hyperinsulinemic-euglycemic clamp in SD rats, the hyperglycemic ZDSD group did not demonstrate the expected supply decrease. Although the RBC velocity and SR were unchanged at baseline between the SD and hyperglycemic ZDSD rats, the elevated RBC SO_2_ in the hyperglycemic group suggests a mismatch in O_2_ being supplied by the blood and the demand of the tissue that occurs in absence of alterations in microvascular hemodynamics at rest. This increased SO_2_ may indeed be a result of overperfusion, which could suggest a loss of appropriate oxygen mediated metabolic regulation in skeletal muscle of ZDSD animals among potentially numerous other impairments. The significantly elevated capillary RBC SO_2_ measurements in the ZDSD group was recapitulated in Protocol 2 in this study. In contrast, the measures of RBC SO_2_ in SD rats is in agreement with saturation measurements collected in young healthy SD rats on the gas exchange chamber (Wells et al., 2023). When comparing the SD and ZDSD strains we found that although we altered the [O_2_] in the chamber there were significant differences between SD and ZDSD SO_2_ at baseline during both 7% periods and 2% [O_2_] (Figure 7). This finding in conjunction with the elevated SO_2_ finding in protocol 1 suggests that there is a mismatch between O_2_ supply and O_2_ delivery in skeletal muscle of ZDSD animals.

Local oxygen mediated blood flow regulation can be interrogated using a gas exchange chamber to dynamically alter local tissue O_2_ conditions. Previous works have shown that delivering elevated [O_2_] to the surface of the muscle leads to a decrease in RBC velocity, SR, and decrease in capillary hematocrit in a dynamic fashion with low [O_2_] causing opposite responses in young, healthy SD rats (Ghonaim et al., 2011; Russell McEvoy et al., 2022; Sove et al., 2021; Wells et al., 2023). Prior studies found no significant differences between baseline and during hyperinsulinemic-euglycemic clamp at any set [O_2_] suggesting that fixing the local oxygen concentration in skeletal muscle leads to a blood flow response dependent on local oxygen conditions regardless of the hyperinsulinemic state (Wells et al., 2023). Interestingly, in this study there was an absence of significant RBC velocity and supply rate responses to changes in local [O_2_] during baseline and hyperinsulinemic-euglycemic clamp in the SD rat group. This lack of response was not expected as prior works on younger SD rats demonstrated robust and rapid hemodynamic responses to challenges between 12% and 2% [O_2_] (Ghonaim et al., 2011; Sove et al., 2021; Wells et al., 2023). The RBC velocity and supply rate at 7% [O_2_] during baseline and hyperinsulinemic-euglycemic clamp, approximately 200 μm/s and 10 cells/s respectively, were similar to those reported previously in SD rats under the same O_2_ conditions (Wells et al., 2023). The lack of response to O_2_ has several potential explanations including differences in age of the animal, the profoundly different high-fat high-sugar diets, the proportion of muscle exposed to the imposed [O_2_] changes, and that animals in the present study were sampled following a 10 h overnight fast. This absence of blood flow responses to altered [O_2_] is a novel finding implicating other interacting factors that may influence evaluation of microvascular blood flow that include age, diet, fasted state, and total tissue area exposed to local [O_2_] perturbations.

Blunted hemodynamic responses have been observed in humans and rodent models of T2D both in response to hyperinsulinemia and in response to exercise (Clerk et al., 2007; Frisbee, 2003; Menon et al., 1992; Raitakari et al., 1997; Reynolds et al., 2017). Due to the blunted responses to hyperinsulinemia and exercise previously observed in rodents, we expected to see slower responses with a smaller amplitude change in RBC velocity and supply rate with the modification in local [O_2_]. The RBC velocity did not significantly change over the course of the 4-min oxygen challenge (Figure 8B) which was further demonstrated when considering the second-by-second time transient tracing (Figure 8A). Similarly, RBC supply rate showed no trend in the second-by-second time series in response to [O_2_] changes (Figure 10). The ZDSD RBC supply rate measured at 7% [O_2_] during baseline were similar to the age matched SD rats as well as to values reported in young SD rats (Wells et al., 2023). The reproducibility between animal strains and across age is promising as it informs us that the ZDSD strain initially has baseline capillary hemodynamics similar to those of young SD rats. Additionally, our study shows that there are no significant differences in hemodynamics between the baseline and hyperinsulinemic-euglycemic clamp conditions regardless of the [O_2_] in the SD group. This consistency between baseline and hyperinsulinemic-euglycemic clamp agrees with the results reported in previous studies on young SD rats (Akerstrom et al., 2020; Wells et al., 2023). Prior work with the Zucker and ZDF rats have shown impaired endothelial dependent relaxation of the coronary and mesenteric vascular beds that is exacerbated with increased age (Oltman et al., 2006) and the endothelial dysfunction in ZDF rats is related to increased inflammation and cyclooxygenase-2 derivatives (Vessieres et al., 2013). Additional studies have identified that high cholesterol feeding also causes impairment in endothelial dependent relaxation in both lean and obese ZDF rats (Hoshida et al., 2000). The SD and ZDSD groups in this study were both fed a high-fat diet that also contained elevated cholesterol; this diet in combination with the animals’ advanced age may both contribute to endothelial dysfunction (Wang et al., 2022) and thus a lack of hemodynamic response to altered [O_2_]. The lack of response to changing local [O_2_] in ZDSD rats is confounding, as we expected high and low O_2_ concentrations to elicit profound microvascular hemodynamic responses that can be measured at the capillary level.

The capillaries rely on upstream arteriolar tone to control blood flow to the capillary bed through vasoconstriction and dilation. One means to evaluate the proportion of capillaries which support tissue perfusion is to classify capillaries based on vessel segments that support continuous, intermittent, or stopped flow. It has been established that 80%–90% of capillaries in skeletal muscle support continuous or intermittent RBC flux at rest (Anderson et al., 1997; Hudlicka et al., 1982; Kindig et al., 2002). Previous work has shown a significant decrease in continuous flow capillaries in prediabetic ZDF compared to age matched controls (Ellis et al., 2010). In our study, there were no significant differences in functional capillary density measurements between any animal groups at baseline or during hyperinsulinemic-euglycemic clamp. When we compared the proportion of continuous, intermittent, and stopped flow capillaries between baseline conditions and those measured during euglycemic clamp there were also no differences. This finding is important as apparent vasodilation in SD rats following hyperinsulinemic clamp does not appear to alter the overall number of capillaries being perfused with continuous flow. This is also an interesting finding as we anticipated a decrease in continuous flow vessels in the hyperglycemic ZDSD group under baseline conditions as observed in previous works in other rat T2D models (Ellis et al., 2010; Padilla et al., 2006) and following hyperinsulinemic-euglycemic clamp.

This study was the first to quantify rat EDL skeletal muscle capillary blood flow in animals older than 14 weeks of age; substantial technical challenges exist when imaging thick tissues like the EDL, which are exacerbated in older, larger animals. Foremost of these challenges related to our approach is the attenuation of incident light intensity within the muscle tissue, where high contrast is needed between RBCs and plasma to obtain accurate RBC SO_2_ measures. Secondly, although the original purpose of using ZDSD rats was to investigate a model that mimics human condition in relation to T2D disease progression, the ZDSD animal strain in our hands did not reliably develop T2D. Some animals that did develop T2D exhibited marked decline in body condition during the weeks leading up to the IVVM protocol. Female ZDSD rats were not evaluated in this study due to the inconsistent reporting of females developing T2D and required a variation in diet regimen to achieve and maintain hyperglycemia (Gonzalez et al., 2014; Hill Gallant et al., 2014). Future works should address this variation in diet regimen required to obtain hyperglycemic female ZDSD rats. The glucose infusion rate to achieve hyperinsulinemic-euglycemic clamp is an indirect measure of insulin sensitivity. During IVVM tail blood glucose sampling we qualitatively noted that the ZDSD rats had poor peripheral perfusion at the distal portion of the tail compared to SD rats, this observation was made during data collection for both protocols. As a result of these differences in blood flow, we sought to examine the consistency between the peripheral and central blood glucose measures. For comparison, we measured arterial blood glucose at a lower frequency; it is not feasible to measure arterial samples frequently due to the volume of blood required. There was a slight discrepancy between the blood glucose measured from the tail and the arterial sample, anecdotally reinforcing the observation that peripheral circulation was impaired in the ZDSD group. At euglycemia, glucose measurements from the tail were consistent with arterial samples throughout the hyperinsulinemic-euglycemic clamp capture protocol. A further challenge with hyperinsulinemic-euglycemic clamp is the longer average time required to achieve euglycemia in the hyperglycemic ZDSD rats compared to the normoglycemic ZDSD and SD groups, which results in the hyperglycemic group receiving additional insulin, fluid, and anaesthetic. However, the cardiovascular state of animals was stable throughout the clamp protocol, and other IVVM studies in rodents have imaged animals for similar or longer durations without complication (Bateman et al., 2001; Bateman et al., 2008).

Further investigation into the level of vasculature that is impacted by O_2_ changes would be warranted with respect to overall muscle thickness and size. The gas exchange chamber has been used extensively with young SD rats and previous work has provided mathematical models of the depth that O_2_ perturbations are expected to penetrate into the tissue with a full gas exchange chamber (Ghonaim et al., 2013) and with micro-outlets (Ghonaim et al., 2013; Ghonaim et al., 2011; Sove et al., 2021). These studies predict that the GEC is able to alter tissue PO_2_ up to ∼100 µm from the tissue surface with the most profound changes in RBC SO_2_ being closest to the surface and progressively decreasing with distance from the exchange membrane (Ghonaim et al., 2013). The difference in muscle thickness and volume is proportional to the body size of the animal and by comparison the relative total volume that is affected by O_2_ perturbations is lower in the 27-week-old SD and ZDSD rats than the 7-week-old, ∼200 g, SD rats that were used previously. The variation in relative tissue volume and level of vasculature impacted by the changes in [O_2_] may provide a partial explanation for the lack of O_2_ response in these larger animals.

In conclusion, this study was the first to quantify skeletal muscle capillary hemodynamics in the Zucker Diabetic Sprague Dawley rat model of type 2 diabetes both under baseline and hyperinsulinemic-euglycemic clamp conditions. The significantly elevated RBC SO_2_ in ZDSD rats measured in this study suggests that there is a mismatch between O_2_ supply and O_2_ demand in skeletal muscle of ZDSD animals. This model of T2D displayed notable increases in blood glucose when fed a high-fat diet accompanied by significant increases in systolic blood pressure compared to their normotensive controls. Our study also demonstrates that there are no significant changes in skeletal muscle hemodynamic response to local O_2_ changes in high-fat fed sexually mature SD and ZDSD animals. This is a confounding finding that suggests that the loss of response to [O_2_] is not constrained to the hyperglycemic state and may be associated with high caloric diets fed to both animal groups, their older age, the relative volume of tissue perturbed using the GEC to elicit a blood flow response from upstream arterioles, or a combination of the above conditions.

## Data Availability

The raw data supporting the conclusions of this article will be made available by the authors, without undue reservation.
